# Effect of transcutaneous electrical acupoint stimulation combined with palonosetron on chemotherapy-induced nausea and vomiting: a single-blind, randomized, controlled trial

**DOI:** 10.1186/s40880-016-0176-1

**Published:** 2017-01-10

**Authors:** Jing Xie, Lei-Hua Chen, Zhou-Yu Ning, Chen-Yue Zhang, Hao Chen, Zhen Chen, Zhi-Qiang Meng, Xiao-Yan Zhu

**Affiliations:** 1 Department of Integrative Oncology, Fudan University Shanghai Cancer Center, 270 Dong’An Road, Shanghai, 200032 P. R. China; 2 Department of Oncology, Shanghai Medical College, Fudan University, 270 Dong’An Road, Shanghai, 200032 P. R. China

**Keywords:** Acupuncture, Electro-acupuncture, Transcutaneous electrical acupoint stimulation, Nausea, Vomiting, Anorexia

## Abstract

**Background:**

Chemotherapy-induced nausea and vomiting adversely affects the quality of life of patients who receive chemotherapy via intravenous infusion or transcatheter arterial chemoembolization (TACE). This study aimed to investigate the clinical effects of transcutaneous electrical acupoint stimulation (TEAS) on nausea and vomiting after TACE.

**Methods:**

A total of 142 patients who received TACE with cisplatin for primary or metastatic liver cancer were assigned to the active-acupuncture (*n* = 72) or placebo-acupuncture (*n* = 70) groups using a covariate-adaptive randomization at a ratio of 1:1. The acupoints Hegu (LI4), Neiguan (P6), and Zusanli (ST36) were stimulated twice daily for 6 days. The effects of TEAS on nausea and vomiting were assessed by using occurrence rate and severity of these symptoms. Anorexia scale and M. D. Anderson Symptom Inventory (MDASI) scores were secondary endpoints and were used to assess the effect of TEAS on patient appetite and quality of life. The safety of the treatments was also monitored.

**Results:**

Between the two groups, the differences in occurrence rates and severities of nausea and vomiting after TACE were not significant (all *P* > 0.05). From the second day after TACE, anorexia scores were significantly lower in the active-acupuncture group than in the placebo-acupuncture group and continued to decrease over time with treatment (all *P* values less than 0.01). On days 0, 1, and 2, the mean MDASI scores for the active-acupuncture group were slightly lower than those for the placebo-acupuncture group, but the differences were not statistically significant (all *P* > 0.05). No significant differences were found between the two groups in the occurrence rate of any adverse event (*P* > 0.05).

**Conclusion:**

TEAS appears to be a safe and effective therapy to relieve patients’ gastrointestinal discomfort after chemotherapy.

*Trial registration* NCT01895010. Registered 21 June 2013

## Background

Transcatheter arterial chemoembolization (TACE) is a palliative treatment of advanced primary and metastatic liver cancer. However, chemotherapy-induced nausea and vomiting (CINV) adversely affects the quality of life (QoL) of patients who undergo this intervention. It is estimated that 70%–80% of cancer patients who received chemotherapy developed CINV, and nausea and vomiting is one of the most debilitating symptoms induced by chemotherapy [1, 2]. Severe vomiting can cause dehydration, electrolyte disturbances, muscle weakness, and weight loss; it may even be life-threatening by inducing acute hemorrhage of the upper digestive tract. CINV drastically lowers QoL and treatment compliance, and it may also affect the success of a specific treatment protocol [3].

Despite improvements in the prevention and treatment of CINV, including the use of type 3 serotonin (5-HT_3_) receptor antagonists [4, 5], there are still potential risks of adverse effects [1, 2]. Therefore, the need for further relief has led to an interest in non-pharmacological therapies [6, 7]. Previous studies have demonstrated the efficacy of acupuncture, which has been used for more than 2500 years in traditional Chinese medicine to cure disease and relieve accompanying symptoms [8–10]. In 1979, acupuncture was approved by the World Health Organization for the treatment of nausea and vomiting resulted from disordered function of the stomach [11]. Some randomized clinical trials have found that acupuncture is an effective palliative treatment of cancer patients, especially for reducing chemotherapy-induced adverse effects such as nausea and vomiting [12]. Transcutaneous electrical acupoint stimulation (TEAS) is an electrical stimulation technique that sends electrical impulses into acupoints through electrodes on the skin surface. TEAS is noninvasive, is easy to use, and does not have obvious adverse effects.

Although noninvasive electro-acupuncture has been shown to benefit patients suffering from CINV [13–15], studies on TEAS combined with anti-emetics, particularly in patients with refractory symptoms or receiving highly emetogenic chemotherapy drugs, are needed to determine if this regimen is also safe and effective to relieve patients’ gastrointestinal discomfort after chemotherapy [16]. In our previous study, we found that electro-stimulation on the acupoint Yongquan (K1) combined with anti-emetics did not result in initial prevention of cisplatin- or oxaliplatin-induced nausea or vomiting [17]. However, it is possible that an extended treatment time and the selection of different acupoints (particularly in the form of combined points) might offer more promising results. Previous studies indicated that stimulation on the acupoint Neiguan (P6) was an effective anti-emetic technique for the prevention of CINV [18, 19], and stimulation on the acupoints Zusanli (ST36) and Hegu (LI4) showed similar results [20–23]. Stimulation on two or more acupoints may improve anti-emetic efficacy, but it lacks the support of clinical trial data. In this study, we chose to stimulate the three acupoints P6, ST36, and LI4 together as a new regimen, which also considered traditional Chinese medicine’s “meridian” theory.

In this study, all participants were diagnosed with primary or metastatic liver cancer, and TACE with cisplatin was considered the optimal treatment. Because cisplatin is highly emetogenic, we used electrical stimulation (active acupuncture vs. placebo acupuncture) combined with a 5-HT_3_ receptor antagonist, palonosetron. Participants were randomly assigned to the active-acupuncture or the placebo-acupuncture group. The acupoints LI4, P6, and ST36 were stimulated twice daily for 6 days. The effects of TEAS on nausea and vomiting, appetite, and QoL were also assessed.

Based on the finding that acupuncture was efficacious for the treatment of CINV, we proposed that electrical stimulation would be effective for patients receiving TACE. Thus, we designed the present single-blind, randomized, controlled trial to examine the safety and efficacy of active acupuncture compared with placebo acupuncture for the treatment of CINV.

## Patients and methods

### Randomization and blinding

All research procedures were approved by the Institutional Review Board (IRB) of Fudan University Shanghai Cancer Center, Shanghai, China (IRB approval number: 1301118-8-1303), and investigations were conducted in accordance with the Consolidated Standards of Reporting Trials statement [24] and the 2010 Standards for Reporting Interventions in Clinical Trials of Acupuncture [25]. The study protocol was registered on ClinicalTrials.gov (Identifier: NCT01895010).

We included all patients at least 18 years old, with primary or metastatic liver cancer and planned to receive cisplatin-based TACE. None of them was receiving other acupuncture therapy. The skin of acupoint site receiving electrical acupoint stimulation must be intact and without visible evidence of injury and can tolerate mild discomfort following electrical stimulation. Exclusion criteria were as follows: (1) combined use of other venous chemotherapy within 5 days after TACE; (2) other confounding factors that may cause nausea and vomiting (such as intestinal obstruction, anorexia, and so on); (3) installing pacemaker; (4) cognitive dysfunction, unable to finish Scale; or (5) TEAS treatment within the past year regardless of indication.

Patients enrolled in this study were assigned into an active-acupuncture group or a placebo-acupuncture group, using covariate-adaptive randomization (or minimization) at a ratio of 1:1. This randomization was used to avoid covariate imbalances resulting from simple randomization, owing to the fact that this was a small study [26]. The study was designed as single-blind, in which the random codes and corresponding treatment measures were known only by the acupuncturist (F.J.). Patients, nurses, researchers, and the principal investigators were unaware of the randomization scheme. Patients who did not receive electrical acupoint stimulation were not told during assignment about the potential responses of the control and electro-acupuncture procedures.

### Patients

This study was conducted in the Department of Integrative Oncology, Fudan University Shanghai Cancer Center, Shanghai, China. All patients received TACE with cisplatin for primary or metastatic liver cancer between January 2012 and December 2014. Investigators explained the research to the patients in detail and answered questions, and patients were informed about the study design, including the use of active and placebo acupuncture, as well as the possible adverse effects of anti-emetic drugs (constipation, headache, and dizziness) and acupuncture treatment (uncomfortable feelings of the skin). Patients were enrolled in the study if they met all the inclusion criteria and did not fall under any exclusion criteria. Enrolled patients were required to maintain close contact with the researchers.

### Interventions

To treat liver lesions, all patients underwent one TACE treatment with cisplatin (60 mg) as the main drug. The TACE procedure took 0.5–1.0 h. The chemotherapy regimen did not include steroids. Before the TACE procedure, all patients received the 5-HT_3_ receptor antagonist palonosetron hydrochloride (0.25 mg, intravenous injection) to prevent vomiting. Then, the patients received the randomized intervention: active or placebo acupuncture. On the first day, patients in the active-acupuncture group received the first electrical stimulation 1–2 h before TACE and another stimulation just after TACE. Acupuncture was continued twice daily for 5 consecutive days, for a total of 6 days.

Patients were asked to adopt a comfortable sitting or supine position. The acupoints were wiped with a moist cotton swab and then connected to the anode and cathode of the electrical stimulation generator (Yao Yang Kang Da Company, Shanghai, China) through an electrode patch placed on the skin surface. Continuous waveform mode was selected, and then the electric current produced continuous stimulation on the acupoints. Stimulation frequency was set at 4 Hz [27], and the default value was set as 10 mA, which was twice the sensory threshold (5 mA). The intensity was adjusted every 10 min to keep the patients comfortable, and its actual value ranged from 7 to 15 mA [27]. Each acupuncture treatment took 30 min.

Acupoints included were as follows. (i) Neiguan (P6): located 6 cm above the transverse crease of the wrist and between the palmaris longus tendon and the tendon of flexor carpi radialis; (ii) Zusanli (ST36): located below the knee, on the anterior tibialis muscle, along the stomach meridian; and (iii) Hegu (LI4): located on the dorsum of the hand, between the first and second metacarpal bones, approximately in the middle of the second metacarpal bone on the radial side.

In the placebo-acupuncture group, the electrodes were placed on the same acupoints and treated for the same duration as in the active-acupuncture group, but the intensity of electric stimulation was 0 mA. During treatment sessions, patients were allowed to watch the operation of the electrical stimulator and view the entire procedure. They could watch the doctor set the acupoint stimulation equipment at 4 Hz and press the start button. Therefore, all patients thought they received real electro-acupuncture.

Chemotherapy, palonosetron, and additional anti-emetics were given by an independent staff physician, who was blinded to the acupuncture treatment. As prescribed by the staff physician, the following rescue anti-emetics were administered: palonosetron (0.25 mg), metoclopramide (10 mg), and steroids (5 mg).

### Outcome measurement

Every day, patients were required to fill out nausea and vomiting diary records to document the severity and frequency of nausea and vomiting. Visual analogue score (VAS) of anorexia is a secondary clinical outcome that measures appetite improvement. VAS ranges from 0 (normal appetite) to 10 (no food intake possible) [28]. In addition, the M. D. Anderson Symptom Inventory (MDASI) [29] was used to assess the effect of CINV on a patient’s QoL. Adverse events were recorded during treatment and at follow-up visits. Some events, such as constipation, headache, and dizziness, could be related to the anti-emetic drugs. Other events, such as tingling, pain, or redness where the electrode contacted the skin, could be related to acupuncture treatment.

### Statistical analysis

Statistical analysis was performed by a statistician from the Department of Statistics, Fudan University Shanghai Cancer Center who was blinded to treatment allocation. Data analysis of baseline characteristics was based on the intention-to-treat population, which included all randomly assigned patients. Baseline demographic and clinical variables of the two groups were compared using Student’s *t* test (continuous variables) or the Chi square test (categorical variables). Efficacy analyses were based on observed patients who completed both baseline and endpoint evaluation. To determine clinical response, the primary outcome variable was analyzed using the Chi square test. Secondary outcome variables, including anorexia VAS and MDASI score, were analyzed in the form of a descriptive comparison using Student’s *t* test. For adverse events, we compared the occurrence rates using Fisher’s exact test. *P* values less than 0.05 (two-sided) were considered statistically significant. All analyses were performed using SPSS 22.0 software (SPSS Inc., Chicago, IL, USA).

## Results

### Baseline characteristics and participants

**Fig. 1:**
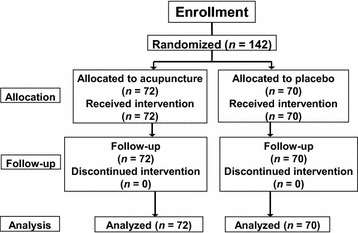
Flow chart of patient inclusion

Of 164 patients with liver cancer who received TACE at our institution between January 2012 and December 2014, 22 either did not meet the inclusion criteria or did not consent to participate. The study was conducted according to the protocol, and the remaining 142 patients completed the study. These 142 patients were randomly assigned to the active-acupuncture treatment group (*n* = 72) or the placebo-acupuncture group (*n* = 70) (Fig. 1). No significant differences were observed between the two groups in terms of baseline characteristics (Table 1).

**Table 1:** Baseline demographic and clinical characteristics of patients with nausea and vomiting after treated with transcatheter arterial chemoembolization (TACE)

Characteristic	Placebo-acupuncture group	Active-acupuncture group	*P*
	(*n* = 70)	(*n* = 72)	
Age (years)^a^	57.5 (30–75)	55.4 (41–77)	1.000
Sex			0.768
Men	47 (67.1)	50 (69.4)	
Women	23 (32.9)	22 (30.6)	
Diagnosis			0.492
Primary liver cancer	15 (21.4)	19 (26.4)	
Metastatic liver cancer	55 (78.6)	53 (73.6)	
Chemotherapy			1.000
Cisplatin-based regimen	70 (100)	72 (100)	
Non-cisplatin-based regimen	0 (0)	0 (0)	
VAS of anorexia^b^	1.50 ± 1.67	1.79 ± 1.82	0.321

*VAS* visual analogue score

^a^These data are presented as median followed by range in parentheses

^b^These values are presented as mean ± standard deviation; other values are presented as number of patients followed by percentage in parentheses

### Efficacy

**Fig. 2:**
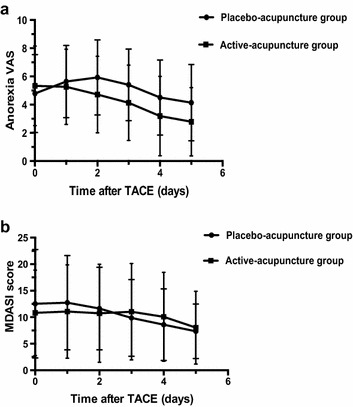
Comparison between active-acupuncture and placebo-acupuncture groups. **a** anorexia VAS; **b** MDASI score over time. *TACE* transcatheter arterial chemoembolization; *VAS* visual analogue score; *MDASI* M. D. Anderson Symptom Inventory

Changes in occurrence rate and severity of nausea and vomiting from the first acupuncture treatment over time are illustrated in Tables 2 and 3. Although fewer patients suffered from nausea and vomiting on days 0, 1, 2, 4, and 5 in the active-acupuncture group than in the placebo-acupuncture group, the difference was not significant (all *P* > 0.05) (Fig. 2). On days 0, 2, and 4, we observed fewer patients with severe or intolerable vomiting in the active-acupuncture group than in the placebo-acupuncture group; and on days 0 and 2, we observed fewer patients with severe to intolerable nausea in the active-acupuncture group than in the placebo-acupuncture group. However, the differences were not statistically significant (all *P* > 0.05).

**Table 2:** Comparison between active-acupuncture and placebo-acupuncture groups in occurrence rate and severity of vomiting

Time after TACE (days)	Patients with vomiting [cases (%)]		*P*	Severity of vomiting [cases (%)]						*P*
				Placebo-acupuncture group			Active-acupuncture group			
	Placebo-acupuncture group	Active-acupu-ncture group		Degree 1	Degree 2	Degree 3	Degree 1	Degree 2	Degree 3	
0	28 (40.0)	23 (31.9)	0.317	42 (60.0)	16 (22.9)	12 (17.1)	49 (68.0)	13 (18.1)	10 (13.9)	0.606
1	25 (35.7)	22 (30.6)	0.514	45 (64.3)	17 (24.3)	8 (11.4)	50 (69.4)	14 (19.4)	8 (11.1)	0.769
2	29 (41.4)	26 (36.1)	0.516	41 (58.6)	20 (28.6)	9 (12.9)	46 (63.9)	18 (25.0)	8 (11.1)	0.809
3	22 (31.4)	26 (36.1)	0.556	48 (68.6)	18 (25.7)	4 (5.7)	46 (63.9)	16 (22.2)	10 (13.9)	0.259
4	16 (22.9)	12 (16.7)	0.354	54 (77.1)	12 (17.1)	4 (5.7)	60 (83.3)	10 (13.9)	2 (2.8)	0.567
5	13 (18.6)	10 (13.9)	0.449	57 (81.4)	11 (15.7)	2 (2.9)	62 (86.1)	8 (11.1)	2 (2.8)	0.721

Degree 1: no vomiting; degree 2: very mild to moderate vomiting; Degree 3: Severe to intolerable vomiting

**Table 3:** Comparison between active-acupuncture and placebo-acupuncture groups in occurrence rate and severity of nausea

Time after TACE (days)	Patients with nausea [cases (%)]		*P*	Severity of nausea [cases (%)]						*P*
				Placebo-acupuncture group			Active-acupuncture group			
	Placebo-acupuncture group	Acupu-ncture group		Degree 1	Degree 2	Degree 3	Degree 1	Degree 2	Degree 3	
0	34 (48.6)	26 (36.1)	0.133	36 (51.4)	22 (31.4)	12 (17.1)	46 (63.9)	19 (26.4)	7 (9.7)	0.256
1	30 (42.9)	26 (36.1)	0.411	40 (57.1)	20 (28.6)	10 (14.3)	46 (63.9)	15 (20.8)	11 (15.3)	0.562
2	30 (42.9)	28 (38.9)	0.631	40 (57.1)	18 (25.7)	12 (17.1)	44 (61.1)	21 (29.2)	7 (9.7)	0.426
3	21 (30.0)	22 (30.1)	0.943	49 (70.0)	15 (21.4)	6 (8.6)	50 (69.4)	16 (22.2)	6 (8.3)	0.993
4	19 (27.1)	17 (23.6)	0.629	51 (72.9)	16 (22.9)	3 (4.3)	55 (76.4)	13 (18.1)	4 (5.6)	0.750
5	14 (20.0)	13 (18.0)	0.768	56 (80.0)	12 (17.1)	2 (2.9)	59 (81.9)	9 (12.5)	4 (5.6)	0.564

Degree1: No nausea; degree 2: very mild to moderate nausea; degree 3: severe to intolerable nausea

*TACE* transcatheter arterial chemoembolization

The mean anorexia VAS increased after TACE treatment until the third treatment of nausea and vomiting after TACE in the placebo-acupuncture group. However, in the active-acupuncture group, the anorexia VAS began to decrease after the first day and continued to decrease with treatment over time. From the second day after TACE, the anorexia VAS was significantly lower in the active-acupuncture group than in the placebo-acupuncture group (*P* = 0.008, 0. 004, 0.005, and 0.002 on days 2, 3, 4, and 5, respectively; Fig. 2).

Patients rated themselves on the MDASI to evaluate the effect on QoL and activities in daily living. On days 0, 1, and 2, the mean MDASI score for the active-acupuncture group was slightly lower than that for the placebo-acupuncture group, but the differences were not statistically significant (all *P* > 0.05) (Fig. 2).

### Safety and tolerability

No serious adverse events occurred in either group (Table 4). Two incidents of mild tingling or redness where the electrode contacted the skin were reported in the active-acupuncture group. All patients’ symptoms resolved once the acupuncture treatment was completed. However, to avoid further damage, patients were still told to apply ice and compression within 6 h after the acupuncture treatment. Eight patients in the active-acupuncture group and ten patients in the placebo-acupuncture group complained of mild or moderate constipation. Laxative drugs were administrated to relieve patient symptoms. Five patients in the active-acupuncture group and four patients in the placebo-acupuncture group experienced headache during the treatment, which was most likely an adverse effect of palonosetron. No significant differences in the occurrence rate of any adverse events were found between the two groups (all *P* > 0.05) (Table 4).

**Table 4:** Occurrence rate of adverse events in the active-acupuncture and placebo-acupuncture groups

	Occurrence rate [cases (%)]		
Adverse event	Placebo-acupuncture group	Active-acupuncture group	*P*
Constipation	10 (14.2)	8 (11.1)	0.570
Headache	4 (5.7)	5 (6.9)	1.000
Mild tingling or redness of the contact part of the skin	0 (0)	2 (2.8)	0.497

### Credibility of placebo and acupuncture procedures

No significant differences were observed in the credibility rating between the two groups. Thirty percent (21/70) of patients treated with placebo acupuncture thought they had received active acupuncture, whereas 19.4% (14/72) of patients treated with active acupuncture thought they had received placebo treatment (χ^2^ = 2.13, *P* = 0.145).

## Discussion

We found no significant effect of TEAS in reducing CINV or improving QoL. However, electrical stimulation on acupoints LI4, P6, and ST36 did markedly relieve appetite loss from the second day after TACE.

CINV is typically associated with a high level of 5-HT, which activates 5-HT_3_ receptors to stimulate vomiting centers. The introduction of 5-HT_3_ receptor antagonists led to significant improvements in the control of acute nausea and vomiting, and 5-HT_3_ receptor antagonists are now part of the standard regimen. However, a substantial proportion of patients continue to experience both acute and delayed CINV after moderately or highly emetogenic chemotherapy. Additionally, adverse events such as headache, constipation, abdominal pain, and dizziness may cause discomfort to patients [4, 30]. Palonosetron is a highly potent, second-generation, selective 5-HT receptor antagonist. One study showed that palonosetron hydrochloride reduced the occurrence rate of acute nausea and vomiting after highly emetogenic chemotherapy by 59.2% [4]. However, even if such a prophylactic approach is taken, sudden nausea and vomiting may still occur. Furthermore, the effectiveness of palonosetron hydrochloride for delayed vomiting remains unsatisfactory, with a reported occurrence rate of 45.3% at a dosage of 0.25 mg and 48.0% at a dosage of 0.75 mg [4]. Repeated administration did not improve results but did increase potential adverse effects and medical expenses. Therefore, to achieve better anti-emetic effects, palonosetron should be combined with other treatment approaches.

Electro-acupuncture was found to be effective in treating moderate CINV [31], with needles inserted into acupoints on the body, which were then attached to a device that generates continuous electric pulses using small clips. Electro-acupuncture might result in complications such as needle syncope, pain, and organ puncture. Furthermore, traditional electro-acupuncture using needles is not always convenient or available in all centers. TEAS is a novel and noninvasive method of acupuncture that has a similar mechanism of action to electro-acupuncture and is considered a safe and effective treatment.

In our study, all patients were successfully blinded and completed the entire study. The credibility rating in the two groups did not differ significantly. Acute CINV usually occurs within the first 24 h after drug administration, and most emetic chemotherapy drugs can trigger nausea and vomiting within 1 or 2 h after drug administration. In contrast, delayed CINV occurs 24 h after drug administration and may last for 6–7 days. Our present study suggested that electrical stimulation on acupoints, combined with 5-HT_3_ receptor antagonists, could slightly reduce the occurrence rate of nausea and vomiting on day 0 and subsequent days (except on day 3), but the therapeutic efficacy was not significant compared with that of the placebo treatment (*P* > 0.05). Similarly, no significant differences were observed between the two groups with regard to the degree of nausea or vomiting on days 0–5. This indicates that electro-stimulation of the combined acupoints was not efficacious in augmenting the anti-emetic effects of 5-HT_3_ receptor antagonists in preventing acute or delayed CINV.

One possible reason why the treatment of more acupoints did not increase the anti-emetic effects more than a regimen that treated only a single acupoint may be that most of the studies reported of these single-acupoint regimens were based on electro-acupuncture with the insertion of needles into the skin, rather than TEAS, which was used in our study. According to a meta-analysis by Ezzo et al. [16], acupressure or surface electro-stimulation may have weaker effects than electro-acupuncture with needles. However, to our knowledge, the electro-stimulation mechanism of multiple acupoints remains unclear, and related fundamental research is needed.

Although our study did not reach its primary endpoint, TEAS was shown to be effective in recovering patient appetites after chemotherapy, as indicated by the significant decrease of the anorexia VAS. The effect was observed as early as the first day after TACE, and the decrease of VAS was greater in the active-acupuncture group than in the placebo-acupuncture group. These results were in agreement with previous studies that demonstrated the therapeutic effects of acupuncture in patients with chemotherapy- or radiotherapy-induced anorexia [32–34]. Acupuncture may stimulate the stomach’s peristalsis and rapidly empty its contents, thus strengthening gastrointestinal motility and resulting in a sense of hunger [35]. This is the most effective way to rapidly improve the appetite; some patients even have a sense of hunger just 1 min after acupuncture. During the early stage of nausea, loss of appetite and feelings of discomfort are the major clinical manifestations. Based on our findings and those of previous reports, we think that a rapid improvement of appetite brought about by TEAS indicates an early sign of recovered gastrointestinal function. Furthermore, a rapid improvement of appetite can help improve patients’ entire recovery process, by reducing sodium electrolyte and energy metabolism disorders, by resulting in weight gain through an increase in food intake, and by improving the emotional state of patients and family members.

The frequency of adverse events in both groups was similar, and only two TEAS-treated patients complained of discomfort at the electrode patch site, which might be related to the acupuncture treatment. Therefore, the treatment was well tolerated and safe.

In summary, our study used combined acupoints P6, LI4, and ST36 rather than a single acupoint to treat CNIV. Although the occurrence rates of nausea and vomiting did not significantly decrease for patients with primary liver cancer who were treated with TACE, the anorexia VAS was significantly lower in the active-acupuncture group than in the placebo-acupuncture group, starting from the second day after TACE. The acupuncture treatment is low in cost, has few adverse reactions, and is simple to administer. Acupuncture may be an optional adjunctive treatment for palliative care; further studies using TEAS are warranted.

## Abbreviations

5-HT: 5-Hydroxytryptamine
QoL: quality of life
TACE: transcatheter arterial chemoembolization
TEAS: transcutaneous electrical acupoint stimulation
CINV: chemotherapy-induced nausea and vomiting
MDASI: M. D. Anderson Symptom Inventory
